# Self-adaptive Bioinspired Hummingbird-wing Stimulated Triboelectric Nanogenerators

**DOI:** 10.1038/s41598-017-17453-4

**Published:** 2017-12-07

**Authors:** Abdelsalam Ahmed, Islam Hassan, Peiyi Song, Mohamed Gamaleldin, Ali Radhi, Nishtha Panwar, Swee Chuan Tjin, Ahmed Y. Desoky, David Sinton, Ken-Tye Yong, Jean Zu

**Affiliations:** 1 NanoGenerators and NanoEngineering Laboratory, School of Mechanical & Industrial Engineering, University of Toronto, Toronto, ON M5S 3G8 Canada; 2 Design and Production Engineering Department, Faculty of Engineering, Ain Shams University, Cairo, 11535 Egypt; 3 School of Electrical and Electronic Engineering, Nanyang Technological University, Singapore, 639798 Singapore; 4 Electrical & Computer Engineering, Faculty of Engineering and Architectural Science, Ryerson University, Toronto, Canada; 5 School of Mechanical & Industrial Engineering, University of Toronto, Toronto, ON M5S 3G8 Canada; 6 Department of Chemistry, University of Waterloo, Waterloo, ON N2L3G1 Canada; 7 Department of Chemistry, Faculty of Science, University of Hail, Hail, Saudi Arabia; 8 Schaefer School of Engineering & Science, Stevens Institute of Technology, Hoboken, NJ 07030 USA

## Abstract

Bio-inspired technologies have remarkable potential for energy harvesting from clean and sustainable energy sources. Inspired by the hummingbird-wing structure, we propose a shape-adaptive, lightweight triboelectric nanogenerator (TENG) designed to exploit the unique flutter mechanics of the hummingbird for small-scale wind energy harvesting. The flutter is confined between two surfaces for contact electrification upon oscillation. We investigate the flutter mechanics on multiple contact surfaces with several free-standing and lightweight electrification designs. The flutter driven-TENGs are deposited on simplified wing designs to match the electrical performance with variations in wind speed. The hummingbird TENG (H-TENG) device weighed 10 g, making it one of the lightest TENG harvesters in the literature. With a six TENG network, the hybrid design attained a 1.5 W m^−2^ peak electrical output at 7.5 m/s wind speed with an approximately linear increase in charge rate with the increased number of TENG harvesters. We demonstrate the ability of the H-TENG networks to operate Internet of Things (IoT) devices from sustainable and renewable energy sources.

## Introduction

Energy harvesting has been an increasingly desirable field of research and progress for the past two decades due to its reliability, cost-efficiency and sustainability^1^. External energy sources like solar, wind, vibration, acoustic, and thermal are in perpetual development to fully actualize sustainable energy farms in the near future^2,3^. To that end, the role of green technologies is fundamental in minimizing dependence on depleting energy sources and their ensuing environmental impact^4,5^. Wind energy is regarded as one of the most opportune sources of clean and sustainable energy^6,7^ for functional energy harvesting against elaborate conditions^8,9^. Other energy harvesting sources have adopted wind energy techniques of rotary/turbine configurations due to the established knowledge from the wind energy harvesting sector^10,11^, indicating its dominance in development and reliability for projected large-scale power plants^12,13^. However, the current standards of wind energy techniques are not without environmental consequences. Multiple studies have stressed the significant environmental impacts of existing wind turbines on local and regional climates^14^. Moreover, the complex, bulky and expensive wind turbines are hardly employed for small systems or personal devices. To counter such negative effect, research on miniature or smaller-scale energy harvesters has evolved rapidly in the past decade^1^. Their conceptual simplicity, higher cost-effectiveness, and mobility in various scientific fields elicit minimal interference upon ecosystems^15,16^. The current field of microscale energy harvesting is still in its infancy, and is in a growing need for enhanced and innovative designs towards adaptive and sustainable energy conversion prospects.

Bio-inspired and biomimetic engineering has illustrated strong potential in enhanced design performance in multiple scientific fields due to unique Multiphysics within the hierarchy of biological structures and organisms^17,18^. Numerous studies adopted biomimicry in solar cell harvesting, mainly by photosynthesis imitations^19,20^, such as in enhanced flight of miniature unmanned vehicles (MUVs)^21,22^. The work flight path of certain creatures (like birds and insects) were investigated to examine their aerodynamics and flight kinematics^23,24^. The hummingbird has been a subject of interest for quite some time due to its sustained hovering and unique wing-stroke maneuvers^25,26^. The aerodynamics of such birds induce vortices over the wing while the wing segments suffer from high temporal deformation patterns during flights in low-wind force scales^27,28^. Recently, the knowledge obtained from the newly developed TENGs^29–31^ has been applied in bio-inspired and biomimetic engineering. TENGs utilize contact electrification and electrostatic induction to convert external stimuli to electrical current^32,33^ for air pollution cleaning^34^, wind energy harvesting^35^, water energy harvesting^36,37^, self-powered machine interfacing^38^, micro/nano-system actuating^39^, etc. TENGs are desirable due to their low-mass density, simple fabrication^40,41^, high-transferability, environmental friendly^42^ and adaptive modes of operation^43,44^. In a bio-inspired TENG model, researchers developed a wireless sensor network (WSN) for enhanced hydrokinetic energy conversion from water-contacted TENGs using a duck-shaped harvester for superior stability^35^. In another example, TENGs were arranged in forest-like arrays of lawn on rooftops to exploit upcoming wind at a multitude of force scales^45^. However, the TENGs have yet to be fully integrated with a bio-inspired design mechanism for enhanced adaptivity and reconfigurability against highly fluctuating wind flow, which could allow the bio-mimetic TENGs to be used in more applications.

In the present work, we investigate a fabricated hummingbird wing fitted with TENGs for reconfigurability and performance against established conditions. The developed design is aimed to shift through weak and strong winds in multiple directions in the simulated environment. The work examines the flapping (Strokes) of the synthesized wings on a contact surface as a contact triboelectric mode. A second mode is introduced by flapping flag objects within the wings to enhance the energy harvesting mechanism from hummingbird mimicry. We have developed a novel, lightweight TENG which harvests wind energy from the environment as well as from human breath and generates power for self-powered applications. Because of its lightweight and extremely simple structure, it has great application prospects for fabricating sustainable wind energy harvesters. We also report high-energy density for one of the lightest TENG harvester with a mass of 10 g, made possible by the bio-inspired hummingbird TENG wing. Moreover, the H-TENG has the advantage of harvesting wind energy from different directions and different angles of attack. Electrical and fluid analyses are incorporated to capture the interplay dependence between the two disciplines during the upstroke and downstroke phase motions of the wing. The harvested energy can be either stored in battery packs or directly integrated with devices. Such characteristics precisely define micro-scale sensing and small-scale wind energy harvesting applications. The developed apparatus is also used to power up humidity, atmospheric pressure and temperature sensors fitted in an Internet of Things IoT infrastructure^46^.

## Experimental

Fabrication of Nanowire Array on FEP Surface: Nanowire array on FEP surface was prepared by a one-step plasma reactive ion etching process as described in detail elsewhere^47^.

Nanopores-Based Aluminum Surface Modification: Relying on 3% mass fraction oxalic acid (H_2_C_2_O_4_) electrolyte, the electrochemical anodization was applied on the aluminum thin film. It was anodized under a bias voltage of 30 V for 5 hours with a platinum plate acting as the cathode. After that, the alumina layer was etched away in a solution of chromic acid (20 g l–1) at 60 °C for 2 h.

Fabrication of hummingbird TENG: To fabricate the hummingbird structure, polycarbonate was selected due to the appropriate mechanical properties of this material to form the wing configuration. Two Wings with 5 × 12 cm^2^ in their areas were fabricated and embedded to create the main unit with three flags each. For each flag, 0.25 mm thick polycarbonate sheet was cut to a wing shape according to the dimensions of the wing. To fabricate the electrodes, 100 nm thick copper layer was deposited using DC sputtering deposition on the surface of the FEP layer which has a thickness of 0.5 mm. Also, Al foils (thickness 0.2 mm) were placed above each face of the wing to act as a tribo and electrode material. Finally, polycarbonate webs were cut and adhered to the inner side of the wing to complete the structure. Then, conical springs with piston-cylinder mechanism were designed, then the mechanism was adhered and fixed at the end of the wing from the head side to allow the bird wing motion.

Electrical Measurement and Characterization: For measuring the electrical output of the device, a programmable electrometer, i.e., Keithley 6514 and a low-noise current preamplifier (Stanford Research System modelSR570) were adopted to monitor the output voltage, current, and charge. Each TENG, as well as their parallel connections, were linked to the measurement system to test various cases. A wind tunnel closed system was also utilized to apply the wind flow to hummingbird TENG (H-TENG) for simulating the flapping motion of the structure generated by wind flow. For testing the hybrid mechanism, different units of H-TENGs were fixed together and experienced flapping motions by the wind. For charging a capacitor, the output of the device was connected to a rectifier that was connected to a capacitor. The Keithley 6514 probes were placed on the capacitor, and the output voltage was measured.

## Results and Discussion

### The design of the H-TENG Harvester

**Figure 1:**
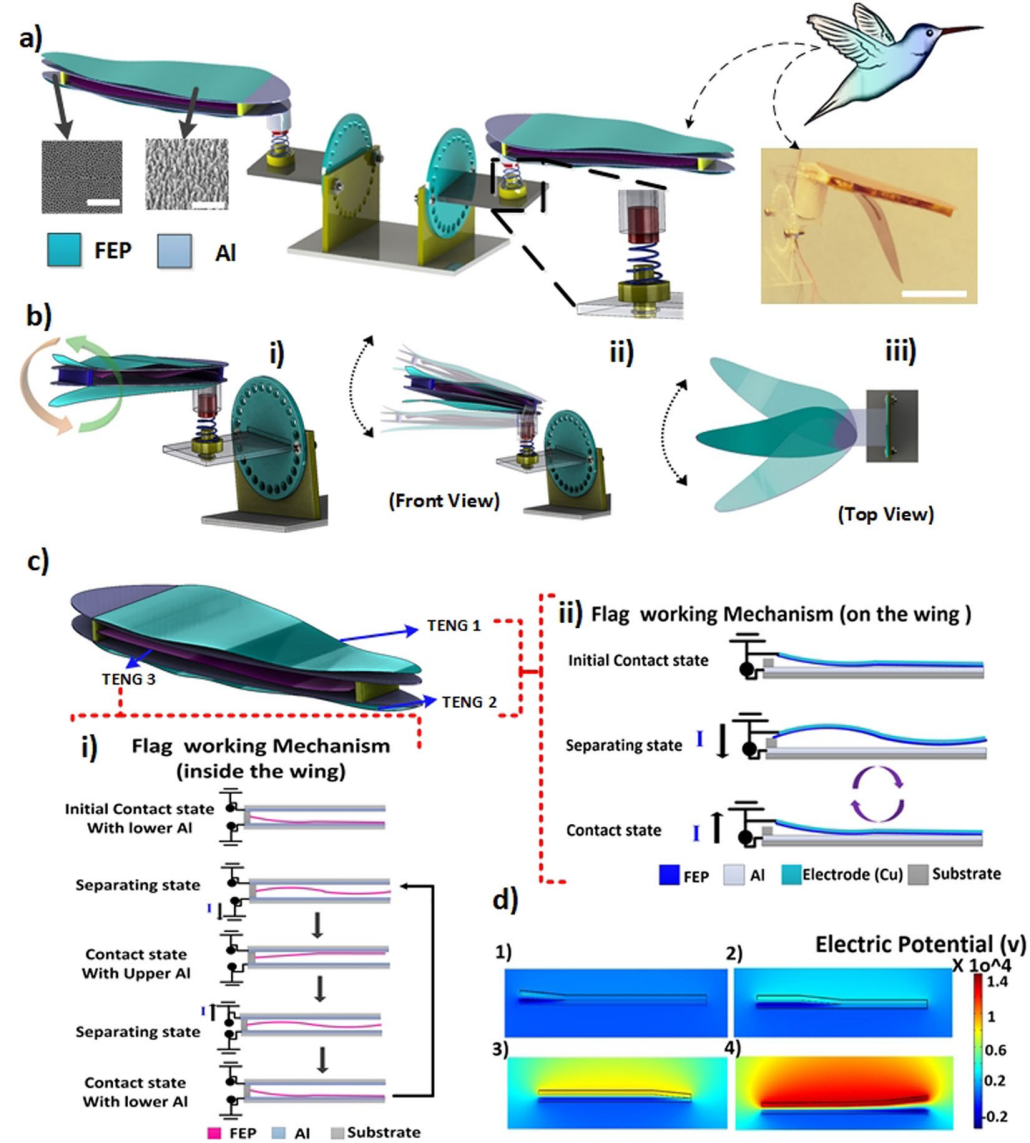
Schematic and experimental structure of a hummingbird wind TENG. (**a**) 3D modeling of the proposed H-TENG wind harvester (with SEM photo at 1 μm scale bar for the Al surface on the left, and SEM image of the FEP polymer nanowires at sale bar is 500 nm on the right), Inset photos represent the real hummingbird bird and the real hummingbird TENG. (**b**) H-TENG kinematics analysis in front and top views (**c**) 3D model of the hummingbird wing with description of the three TENGs configurations; at the top (TENG 1), at the bottom (TENG 2) and inside the wing (TENG 3) (i) Working mechanism of the flag TENG which is placed on the top and bottom of the H-TENG harvester wing. (ii) Working mechanism of the flag inside the wing as a second mode to harvest the mechanical motion resulting from the wind and mimicking the hummingbird flapping motion, (**d**) Potential distribution of the device for different flags using COMSOL.

In this study, a bio-inspired H-TENG design is utilized to simulate the bird wing kinematics for power harvesting and sensing applications. The fabricated harvester induces high-speed wing flutter that captures the commendable flapping-path of this adaptive creature. The H-TENG based wind harvesting system is demonstrated in Fig. 1. The energy generation of the proposed design is majorly governed by two mechanisms: external flag motion and internal single-electrode TENG. The first mechanism utilizes upper and lower flags that flap onto their respective surfaces, creating a high sequence of oscillatory contact-propagation-separation motion. Thus, the total contact area of the two tribo-layers is increased and decreased steadily as a result of wind turbulence. Therefore, an effective contact area strongly dictates the electrical output from the fluttering behavior. Figure 1b shows the images of wing kinematics behavior for the single and double-contact modes (see supporting Movie S1), The H-TENG configuration, shown in Fig. 1c–i, was designed based on these working mechanisms. The H-TENG consists of a metal (Al) and an insulator (Fluorinated Ethylene Propylene, FEP) structure in contact. FEP tends to gain electrons on its surface due to electrification effects and becomes negatively charged. Strong electrostatic attraction ensures adequate contact of the Al with FEP flag under no external flow. When a wind flow is induced, the flag experiences a backward and forward movement resembling a pendulum motion. In the separate state, the electrical charge difference is reduced to zero as no contact is established. In the contact state, the propagation process occurs immediately after contact that causes a sequential process of gradual increase and decrease in contact surface area. To put it simply, a higher electrical potential upon the FEP flag is amplified with a steady increase in the contact surface and vice versa. An alternating current from the Al electrode and FEP flag is generated from the applied potential difference that originates when the flag sequentially approaches the plate.

**Figure 2:**
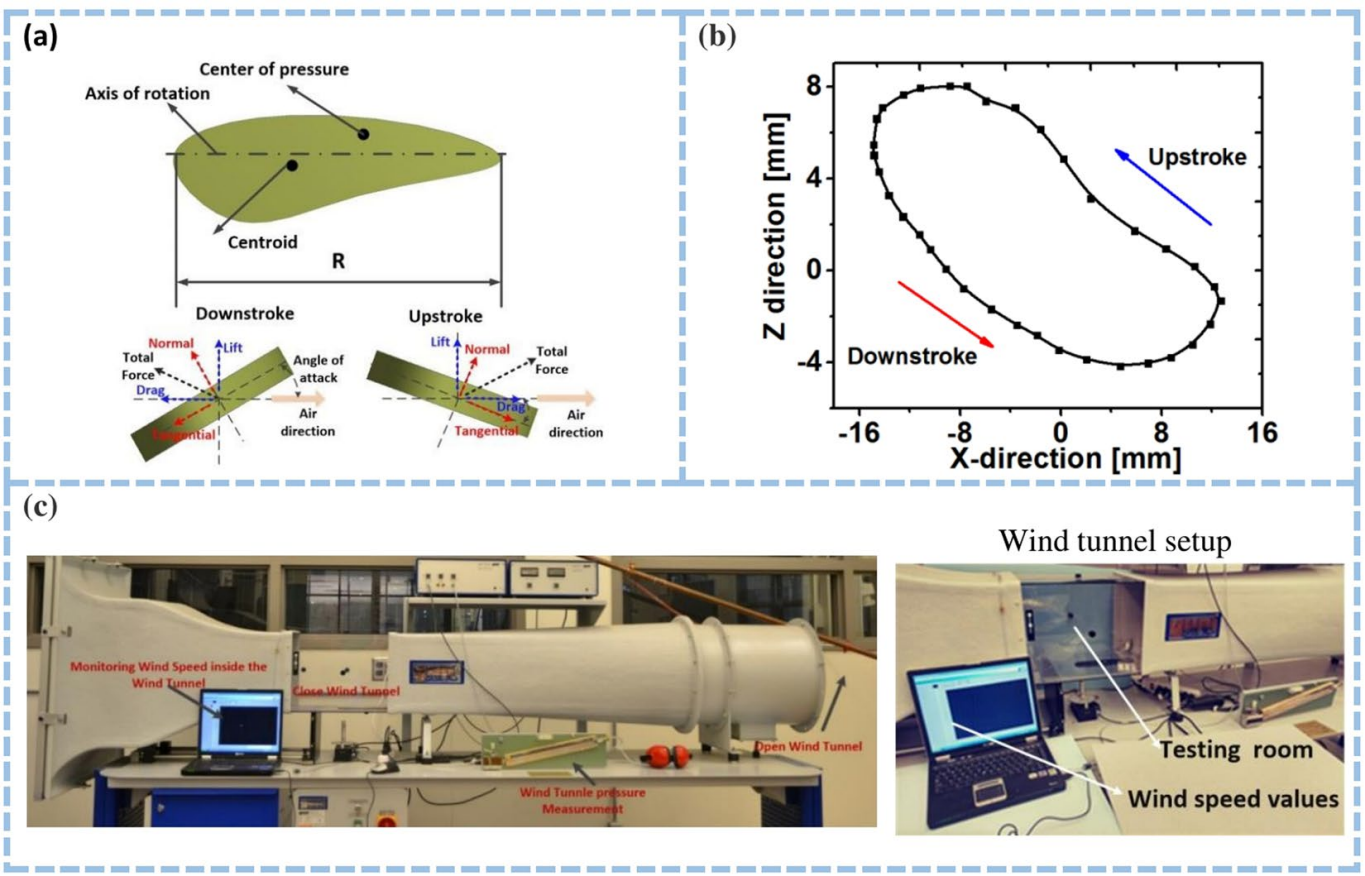
Wing aerodynamics modeling and experimental setup. (**a**) Wing geometry parameters and components of the total aerodynamic force in upstroke and downstroke. (**b**) The averaged trajectory of the wing tip in the XZ-plane. (**c**) Measuring setup inside the closed system wind tunnel while monitoring the wind speed values and the wind tunnel.

**Figure 3:**
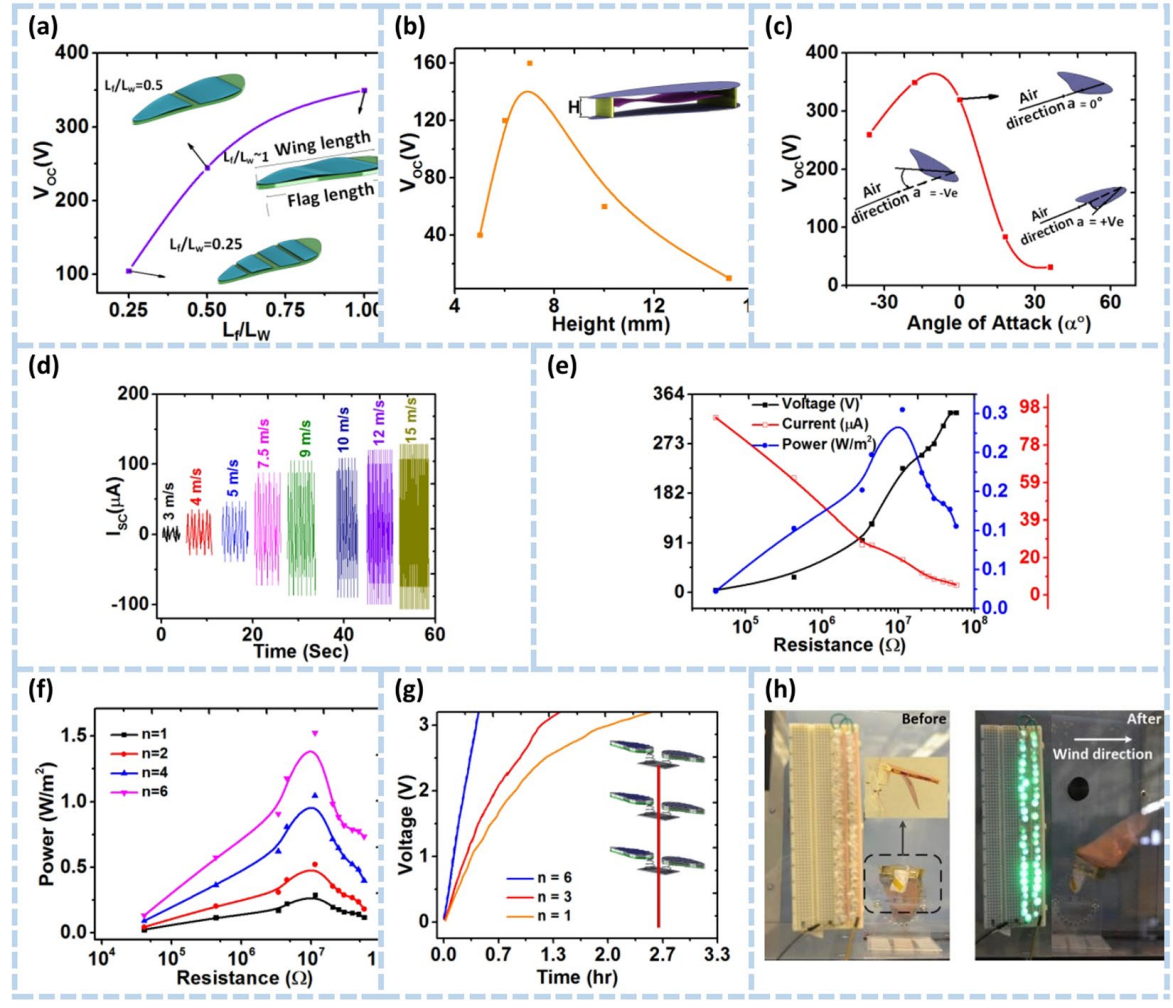
H-TENG characteristic studies. (**a**) Output voltage of the TENG at different flag length ratios. (**b**) The output voltage of the TENG at different heights of the channel inside the wing from 5 mm to 15 mm. (**c**) The output voltage of the TENG at different angles of attack of the channel from −36° to 36°. (**d**) Dependence of the Short-circuit current, I_SC_ of the H-TENG with varying wind speeds from 3 to 15 m/s. (**e**) Load resistance dependency on the current, voltage and power density for one unit of H-TENG. (**f**) Dependence of the power output with a number of units, n (n = 1, 2, 4 and 6) on the resistance load. (**g**) Charging curves of a capacitor (capacity: 1 µF) for energy storage by one, three and six hummingbird TENGs. Inset figure is a tree shape network of hummingbird TENG units for portable wind harvesting applications, (**h**) Image of 50 powered LEDs using one unit of the H-TENG.

**Figure 4:**
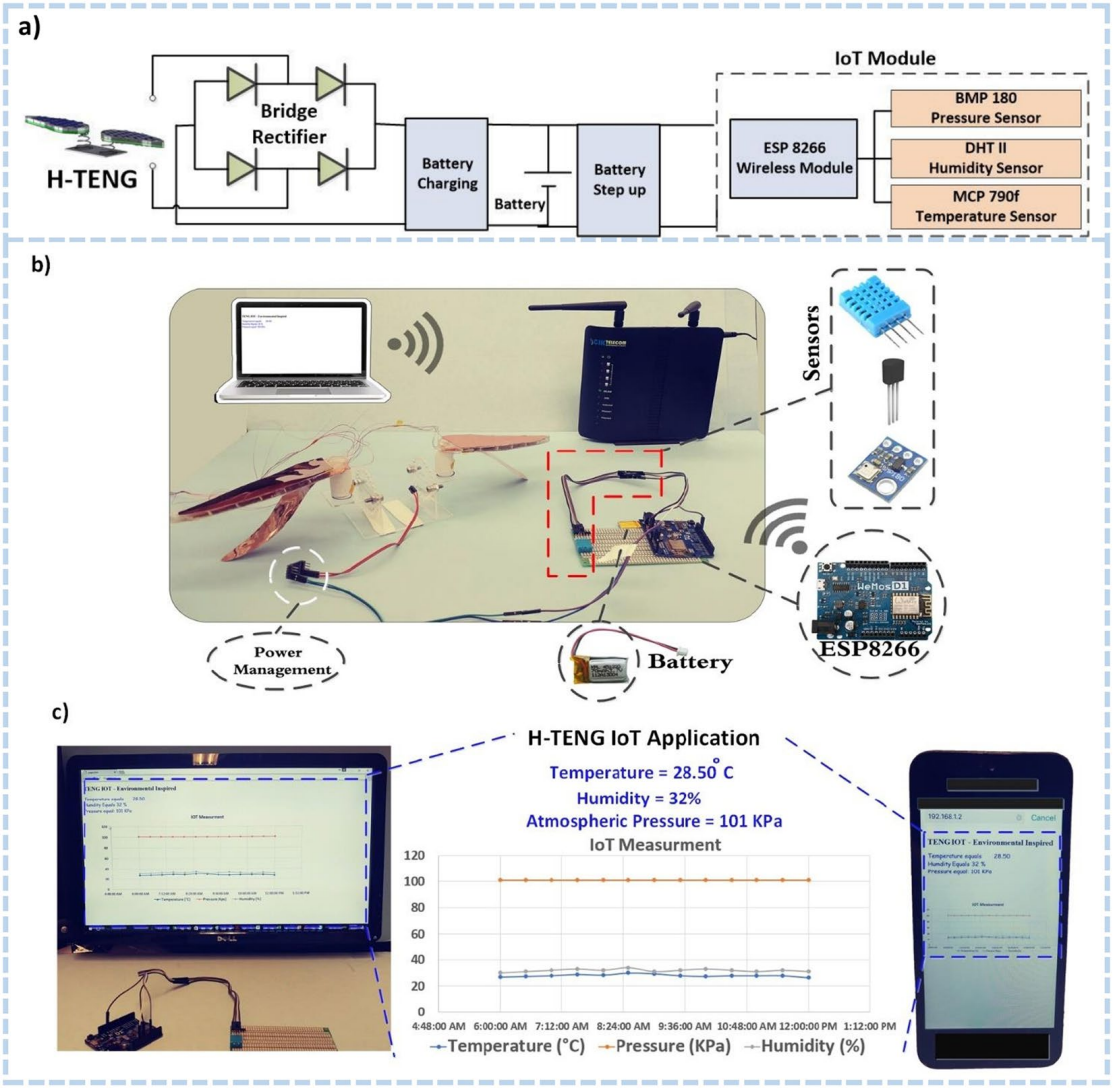
(**a**) A circuit diagram of the self-powered wireless environmental node (pressure-temperature-humidity) for IoT applications enabled by H-TENG. (**b**) The power management circuit, battery, router, environmental sensor node, H-TENG and a wireless module. (**c**) The temperature, pressure, and humidity are transmitted from the wireless sensor node system to a mobile and a computer screen. The inset figure shows the enlarged values of sensor outputs on a web browser in a laptop as its IoT application.

Another wind energy harvesting mechanism, displayed in Fig. 1c–ii, is also investigated in our H-TENG. In this case, electricity is generated from two TENG units embedded within the wing design with a vertical contact-separation TENG mode. Full contact is secured from fractions of the top Al and FEP film such that a triboelectric polarity difference induces triboelectric charges. When the FEP film moves towards the top, the electrical measurements on the two TENGs inside the wing reveal an output current/voltage signal on each TENG as in contact state with the top Al (Fig. 1c–ii). To understand the underlying mechanisms, a computational analysis was conducted on a single H-TENG by finite element method (FEM) in COMSOL software. A two-dimensional schematic flow of the contact-propagation-separation motion with charge distribution simulations is presented in Fig. 1d. Figure 1d–1 shows the initial contact between the flag and plate without external flow, while Fig. 1d–2,3 demonstrates the decrease in contact area during external flow. Finally, Fig. 1d–4 shows the maximum amplitude of fluttering motion phase.

### Aerodynamics modeling and experimental setup

A Multiphysics computational analysis was conducted to evaluate the fluid-solid interactions and the aerodynamic performance of the TENG device. The main force components of the present study are wing translation forces with normal and tangential part F_N__tr_ and F_T__tr_, wing rotation forces F_Nr_ and added wing mass inertia forces F_Na_. Figure 2a illustrates the applied normal and tangential components and their direction, along with their respective relations with drag and lift forces. During the upstroke of the wing, the pressure and velocity vector distribution results are calculated and depicted in Supplementary Figure 2(a), whereas Fig. 2a displays the essential geometric parameters of the wing with wing length R and mean chord length c.

It is worth mentioning that variations in the force occur only with the velocity of the center of pressure UCP at a specified angle of attack α and a given form of the wing. The angle between the wing’s negative x-axis and the UCP velocity vector is what we have defined as the angle of attack as shown in Fig. 2a. When the normal component of the forces F_N_ is set at the z- direction, the positive values are obtained during the downstroke and negative at upstrokes for right hovering case hovering. The tangential component of the force F_T_, which acts in the x-axis, is either positive or zero during hovering. The lift forces F_L_ are attributed to the resultant normal forces within the instantaneous stroke plane, while drag F_D_ is defined as the opposing force to the direction of the instantaneous tip velocity vector. Over on cycle, the lift C_L_ and drag C_D_ coefficients are calculated and portrayed in Supplementary Figure 2(b,c), respectively. The positive dependency of the drag on the vertical forces is represented in Supplementary Figure 2(b) during the first half of the downstroke. The second half of the downstroke shows minor negative/detrimental relations between the two quantities.

Drag was observed to be negative, on the most part, during upstrokes of the wings with small magnitudes. Excluding the vertical-drag forces would not render the lift coefficient C_L_ symmetric between downstroke and upstrokes, as observed in Supplementary Figure 2(d), along with its vertical component, C_L_, Z. A similar, asymmetric trend to the vertical forces is perceived in Supplementary Figure 2(c) for the power coefficient. Additionally, the computational analysis has shown that the ratio of powers exerted between downstroke and upstrokes is about 2.8. The power coefficient value was averaged throughout the cycle magnitudes and was found to be C_P_ ~ 2. Trajectories from cycle-averaged calculations were obtained for the right-wing tip in the XZ plane, where the plot in Fig. 2b represents the deviation from the mean stroke plane. Decomposition of the generated force into aerodynamic lift and drag is provoked from the mean observations of the figure. From Fig. 2a, the aerodynamic lift corresponds to the force perpendicular to the wing translation while aerodynamic drag is attributed to the force opposite to the wing translation. Moreover, as illustrated in Fig. 2c, all measurements were conducted inside a closed wind tunnel. This enabled effortless control of the wind speeds while observation recordings were made.

### System Optimization

The geometrical and material properties of the device were calibrated according to the highest performance achieved by the TENG setup. Initially, the H-TENG is optimized for output results with different triboelectric materials as shown in Supplementary Figure 1(b,c) (see Supplementary Note 1). Moreover, the number of flag strips inversely affects the output voltage as illustrated in Fig. 3a and Supplementary Figure 1(a). Wind cross flow on the FEP flags generates a distinct vibration mode on each flag that causes a chaotic status in contact-separation behavior between the triboelectric layers. The interfacial charge transfer can thus be suppressed by this behavior, leading to a lower output. Furthermore, the aerodynamic energy is converted into electricity by the flag-shaped TENG device by changing contact status on the FEP/Al foil. The output voltage first increases and then decreases as the height of the TENG flag inside the wing is increased (Fig. 3b). The maximum output voltage is attained at a height of 7 mm, and thereafter all subsequent experiments are conducted at 7 mm height between the two wings. This arrangement is selected to ensure effortless and effective switching between the FEP film and Al electrode in contact-separation mode at the fabricated optimum height. As a result, the flag is allowed to harness more wind energy than the other configurations since the mechanical motion and the electrical power output of the H-TENG design is heavily influenced by the angle of attack. The optimum output was achieved at an angle of attack of approximately −18°, as illustrated in Fig. 3c. This would permit the wing to have a higher lift coefficient and a lower drag force. The angle of attack is made adjustable in the current design in order to control the wind direction.

Extremely low and variable motion frequencies are inherent in wind flows. Ultimately, device performance must be investigated intensely against different wind speeds for practical applications. The wind speeds are made to vary from 3–15 m/s to examine a multi-unit H-TENG from low to high wind speeds. The bio-inspired design not only achieves a unique kinematic excitation for superior energy harvesting but also enhances the lightweight limit a TENG device can reach. The H-TENG device weighed just 10 g, illustrating a high potential for large energy harvesting networks with a high-density H-TENG distribution. To our knowledge, our current design is one of the lightest TENG for wind energy harvesting. This holds a promising position for energy efficiency, transferability, and deployment which makes the design more competitive with current state-of-the-art wind energy harvesting technologies. The short-circuit current I_SC_ results are shown in Fig. 3(d) for multiple wind speeds. The output current is notably small at low levels of wind speeds. At higher wind speeds, the measured parameters have a drastic increase in their respective peak amplitudes, suggesting a linear dependence between the current and wind speed. Such performance originates from large aerodynamic forces with lesser contact times between the surfaces. This entails a wide variety of a potential application involving our flag-like TENG for wind speed detection, humidity, and pressure estimations. For wind speed values larger than 9 m/s, the voltage and current do not experience the same dramatic increase when higher speeds dominate the wind flow.

On a single TENG unit, external loads are crucial to voltage, current and power performances of the device. For characterizing the H-TENG outputs under different loads, three TENG flags were made on a single H-TENG unit, connected in parallel with a variable resistor box. The effect of changing the external loads on voltage and current values at a wind speed of 7.5 m/s is captured in Fig. 3e. An exponential increase in voltage is noticed as the resistance increases, where the peak value matches the highest resistance state. However, with an external load of the minimum resistance value, the Ohmic losses impose a disproportionate behavior on current, thus attaining a maximum current output at that minimum. A maximum power output is observed at an external load of 10 MΩ. At this resistance value, a peak power density value of 0.3 W m^−2^ was gained for the overall device. Multi-network integration feasibility was also assessed for the nanogenerators. To investigate the effect of the number of H-TENG units on output power, six identical H-TENG units were fabricated to contain three TENGs each as shown in Fig. 3g. The hybridized system is thus obtained by connecting the units in parallel for subsequent testing at a wind speed of 7.5 m/s. The power generated against load resistances for different numbers of integrated TENG units (n = 1, 2, 4, 6) is shown in Fig. 3f. It was observed that the power peak value would significantly enhance as the number of units is increased up to a maximum peak power of 1.5 W m^−2^ for n = 6. Therefore, the hybrid system of H-TENGs would be an excellent option for wind energy harvesting networks. One such case of network generators is exemplified in Fig. 3g. Simplified attachments of H-TENG unit series can be fitted with wind guides for directional adjustment of oscillating units to accommodate the wind incidence angle to maximize energy harvesting from multi-directional wind flow. Moreover, this adjustment in the angle would minimize external interactions of the devices to alleviate the effects of excessive forces from harsh environmental conditions.

A direct and proportional correlation is derived from between the number of H-TENG harvesters and the charging accumulation rate. A larger number of TENG units is expected to generate triboelectric charges faster with a higher charging accumulation rate. This is substantiated in Fig. 3g, where the TENG peak power output is vastly enhanced with higher harvester numbers. A capacitor with a 1 μF charge rate is affected by the parallel-connected TENG harvester networks, evident from Fig. 3g, with a specified number of units at 7.5 m/s. The same time interval of roughly 0.6 h. Resulted in a voltage of 3.3 V for the chosen capacitor charged by a hybrid system of six parallel units. This further attests to our previous assertion that integrated harvester networks would have a linear-proportional impact on the electrical power output with an elevated number of units. The experimental platform is shown in Fig. 3h and supporting information Movie S2.

### H-TENG based self-powered device for IoT and environmental sensing

From the recent advancement in technologies, Internet of Things IoT has a practically huge social and economic impact around the globe^46^. Owing to the small size and unique self-powered operation ability, the proposed bio-inspired H-TENG has enormous potential to be used for IoT devices as a consistent power supplier or sensor for each device. In this study, we used a wireless temperature sensor (ESP8266 module) with the proposed TENG device to estimate the power harvesting performance for IoT node operation (see supplementary note 2 Methods section). Energy harnessed by the H-TENG device was stored by the rectifier (70 mAh) and a battery charging module, shown in Fig. 4a, which could then be applied to an environmental IoT sensor module for processing and transmission of signals and sensor functions (Fig. 4b). The hybrid system of six TENG devices was connected in parallel and run for 60 mins at a wind speed of 7.5 m/s. Thereafter, the discharged power from the battery unit was used to operate the environmental sensor node for transmitting data at 1-second intervals. Personal computers or mobile devices act as receivers for displaying the data in any web browser by assigning the proper IP into the URL field (see Fig. 4c). This portable device can provide immediate feedback of the environmental conditions, and it can be used to retrieve useful real-time data from inaccessible areas in coastal, high-elevation and suburban locations. Once installed, the self-powered sensors in these devices can readily provide essential information on temperature, pressure, and humidity for environmental monitoring.

## Conclusions

In this paper, a functional TENG is developed for wind energy harvesting through an innovative hummingbird wing-inspired design. The lightweight, low-cost TENGs are fully enclosed within the wing design with a fluttering wing inside to induce contact-separation and free-standing electrification modes with external contact-separation flags for multiple triboelectric material combinations. Mimicking the unique strokes of the hummingbird’s wings, the fluttering of H-TENG can operate with low-frequency wind excitations at harsh conditions. The 10 g lightweight H-TENG device is reported to be one of the lightest TENG device ever made, realized by the bio-inspired design of the hummingbird wings. This provides enormous potential for TENGs to be more competitive than ever in terms of energy efficiency with low mass and low-cost for deployment and installation as opposed to current wind energy technologies. Parallel connections of single TENG units were fabricated in a hybridized wind harvester network. The hybrid system of six H-TENG units is capable of achieving a peak electrical output of 1.5 W m^−2^ at an optimum wind speed of 7.5 m/s. Varying the number of TENG units experienced a linear relation with the output power density and charge rate, making it highly desirable for a multi-modal and more comprehensive wind energy harvesting. Furthermore, an IoT device is powered by the presented nanogenerators for wireless sensor networks and remote operations such as humidity, temperature, and atmospheric pressure sensors.

## Electronic supplementary material

Movie s1

Movie S2

Supporting Information H-TENG
