# Supplementary material for: Self-adaptive Bioinspired Hummingbird-wing Stimulated Triboelectric Nanogenerators
Source: Sci Rep. 2017 Dec 7;7:17143. doi: 10.1038/s41598-017-17453-4 (PMC5719441; doi:10.1038/s41598-017-17453-4)
Supplement: Supplementary file 3 — Supporting Information H-TENG [file 41598_2017_17453_MOESM3_ESM.docx]

**Self-adaptive Bioinspired Hummingbird-wing Stimulated Triboelectric Nanogenerators**

Abdelsalam Ahmed ^1,^ , Islam Hassan^1^**^,^** ^2^, Peiyi Song^3^, Mohammad Gamaleledin ^4^, Ali Radhi ^1^, Nishtha Panwar ^3^, Swee Chuan Tjin ^3^, Ahmed Y. Desoky ^6,7^, David Sinton^5^, Ken-Tye Yong^3^, and Jean Zu ^8^.

**^1^**NanoGenerators and NanoEngineering Laboratory, School of Mechanical & Industrial Engineering, University of Toronto, Toronto, ON, M5S 3G8, Canada.

^2^Design and Production Engineering Department, Faculty of Engineering, Ain Shams University, Cairo, 11535, Egypt.

^3^ School of Electrical and Electronic Engineering, Nanyang Technological University, Singapore 639798, Singapore.

^4^ Electrical & Computer Engineering, Faculty of Engineering and Architectural Science, Ryerson University, Toronto, Canada.

^5^ School of Mechanical & Industrial Engineering, University of Toronto, Toronto, ON, M5S 3G8, Canada.

^6^Department of Chemistry, University of Waterloo, Waterloo, ON, N2L3G1, Canada.

^7^ Department of Chemistry, Faculty of Science, University of Hail, Hail, Saudi Arabia.

^8^ Schaefer School of Engineering & Science, Stevens Institute of Technology, Hoboken, NJ 07030, USA.

**Supplementary Figures**

| (a)  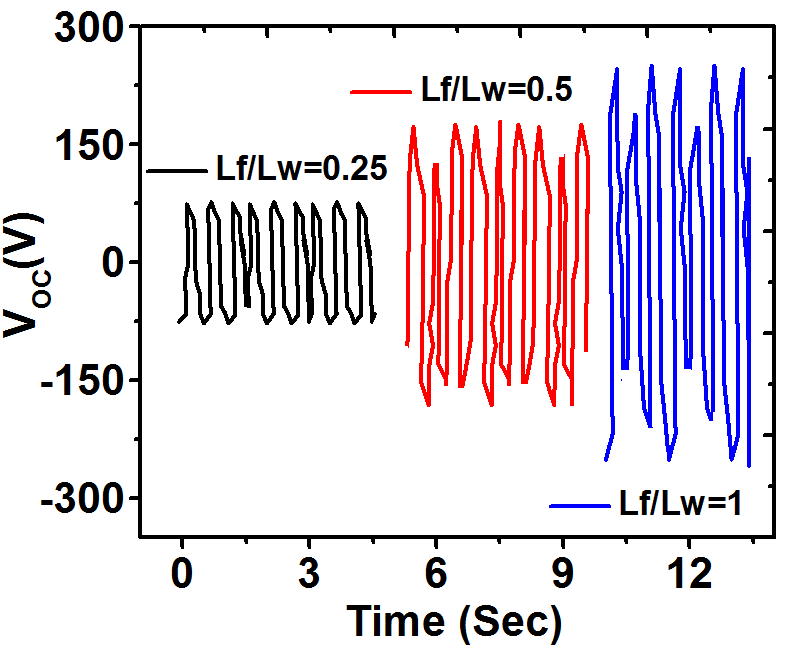 | (b)  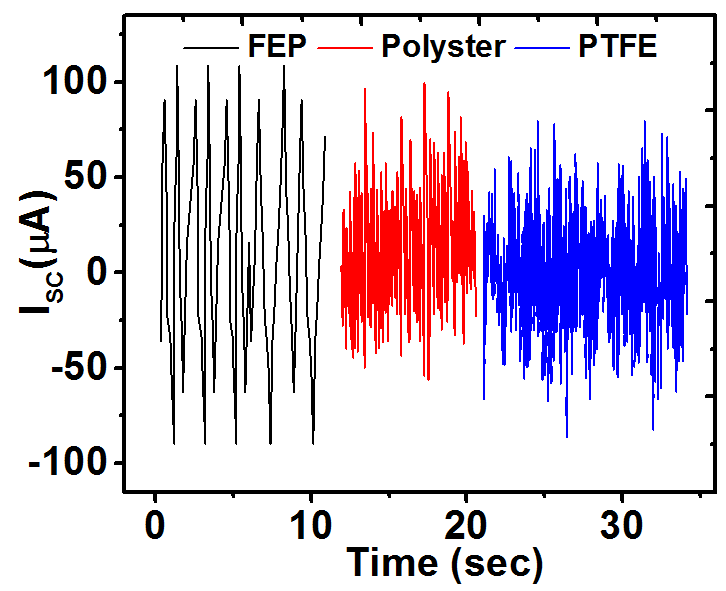 |
| --- | --- |
| (d)  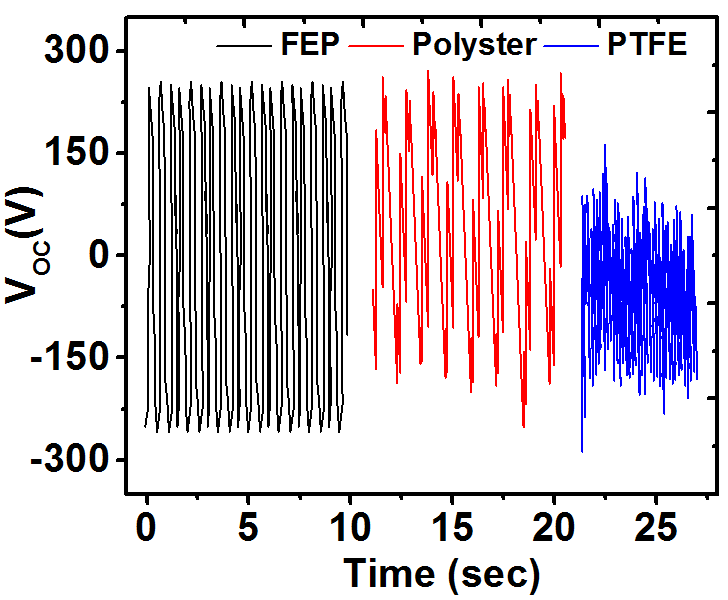 | (d)  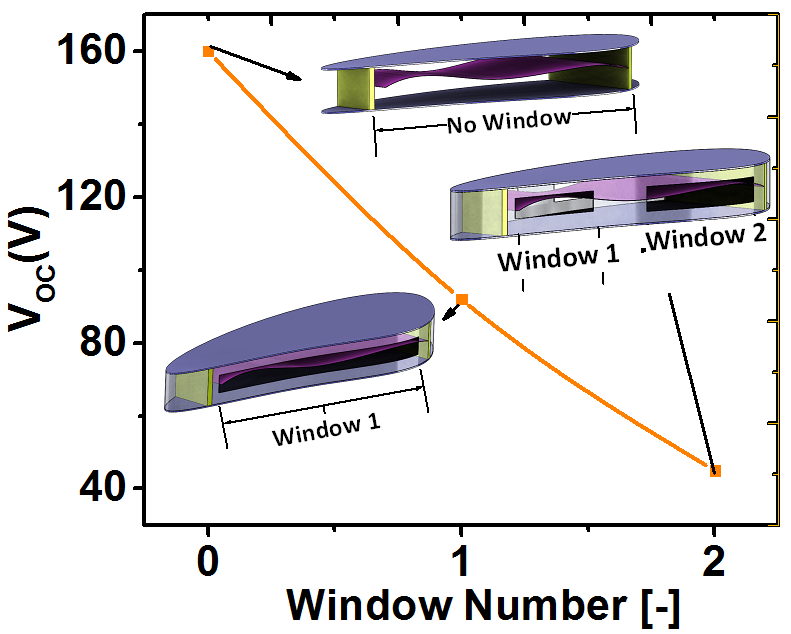 |

**Supplementary Figure 1** (a) Output voltage of the TENG at different flag length ratios. (b) and shot circuit current I_SC_ as a function of time for different tribo materials (FEP, PTFE and Polyester), (c) Open circuit voltage V_OC_ as a function of time for different tribo materials (FEP, PTFE and Polyester). (d) Output voltage of the TENG at different configurations by designing the channel inside the wing with and without windows.

| (a)  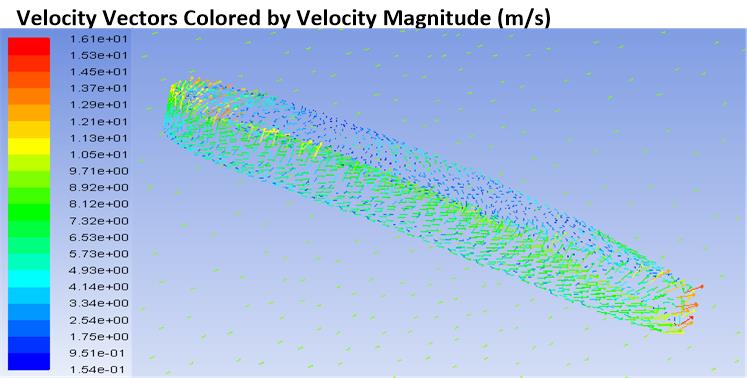  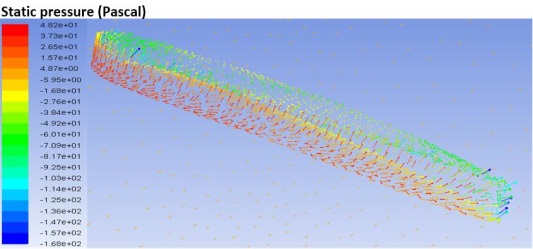 | (b)  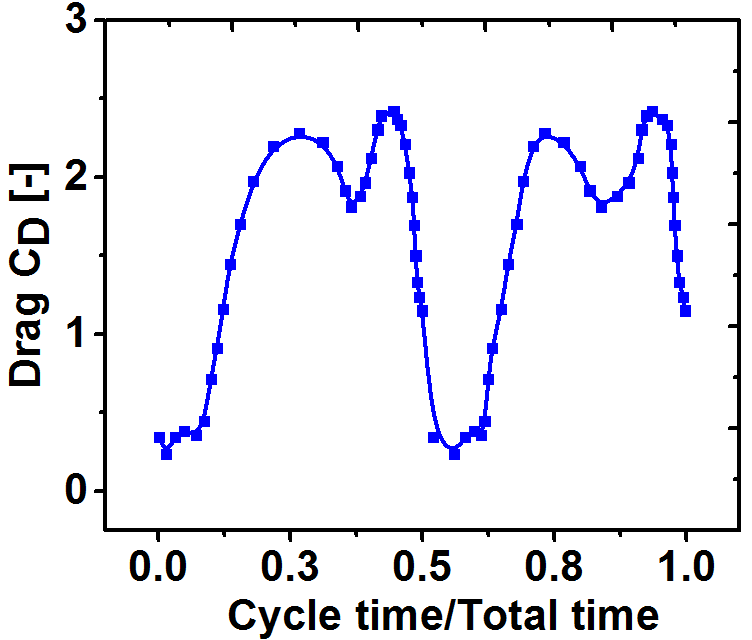 |
| --- | --- |
| (c)  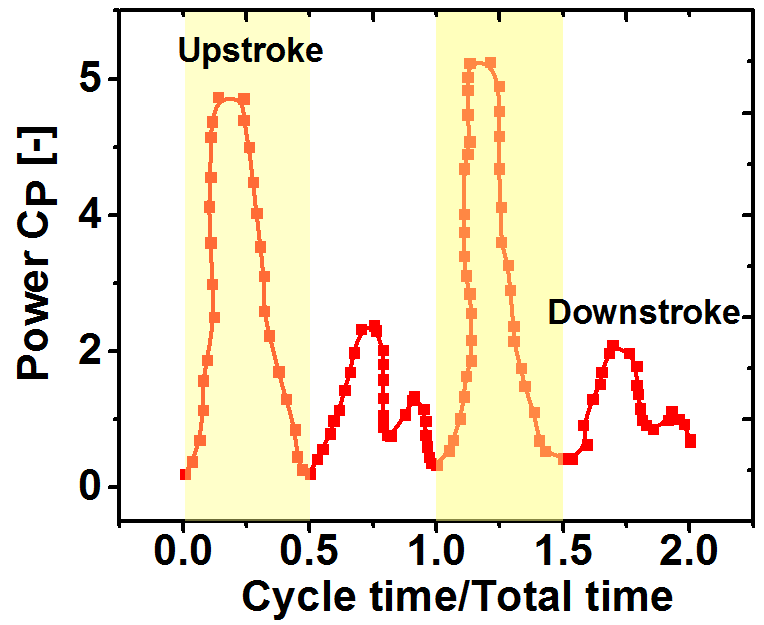 | (d)  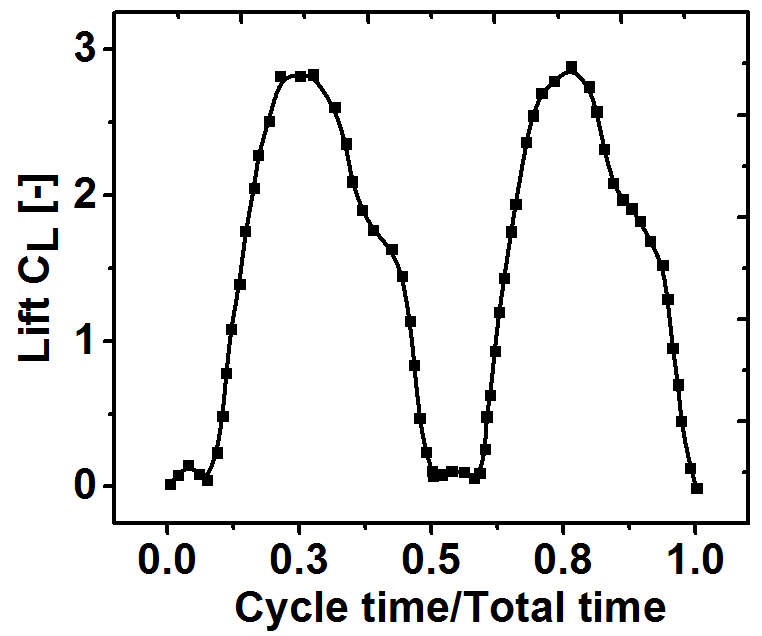 |

**Supplementary Figure 2** (a) Velocity and pressure vector field distributions around the wing at air speed 8 m/s. (b) Predicted Instantaneous drag force coefficient over one applied cycle with computational fluid dynamics (CFD). (c) Predicted Instantaneous power coefficient over two applied cycles with computational fluid dynamics (CFD). (d) Predicted Instantaneous lift force coefficient over one applied cycle with computational fluid dynamics (CFD).

**Supplementary Notes**

**Supplementary Note 1 (Please refer to Supplementary Figure 1)**

The geometrical and material properties of the device were calibrated according to the highest performance achieved by the TENG setup. Initially, the H-TENG is optimized for output results with different flag triboelectric materials as shown in Supplementary Figure 1(b, c). The three material options used in this study are FEP, polytetrafluoroethylene (PTFE) and polyester films. For the FEP/Al TENG setup, the voltage and current output are measured to be the strongest. The open circuit conditions, similar to initial setup, yield a voltage, V_OC_ of 350 V approximately for the FEP/ Al TENG while the short circuit conditions result in a current of about 100 μA at 7.4 m/s wind speed. Secondly, the flag length ratio, defined as flag length Lf / blade length Lw, is an essential factor for further optimization of the H-TENG configuration. The maximum V_OC_ of 350 V was achieved at a flag length ratio of ~1. For another factor that may affect the H-TENG efficiency, three unique designs with multiple formations were additionally considered to the internal wing structure for TENG testing within the wing. The first design comprises of two windows and a cover in the immediate vicinity of the wing. The second design utilizes a large window while the third removes all present windows. The design, shown in Supplementary Figure 1d, has the optimum output configuration when no shield windows are attached

**Supplementary Note2 (Materials and Methods)**

**Experimental Setup for IoT environmental sensing**: In the present study, we applied the IOT concept using an ESP8266 unit. This chip has ultra-light power consumption Wi-Fi, along with a full Transmission Control Protocol/Internet Protocol (TCP/IP) stack and microcontroller. The device requires 2.4 GHz with multiple General-purpose input/output (GPIO) pins. It is supporting Inter-Integrated Circuit (I2C) and Serial Peripheral Interface (SPI). During initialization process of the sensor node, the battery voltage was reduced to about 3 V and an hour was needed to bring the voltage back to 3.3 V. the hummingbird TENG is used to charge the battery for operating it for much time. A lithium battery is charged by the TENG output power, regulated by a power management circuit to achieve power saving operations. The server is downloaded on the ESP8266 chip with internet connection and a designated Internet Protocol (IP). Sensors measurements can be read and uploaded to the client port through Asynchronous JavaScript and XML (AJAX) technology, which is a web technology that stands for Asynchronous JavaScript and XML. AJAX provides us updating sensor`s read section only on the page instead of updating the whole page every interval. The selected environmental node is capable of encompassing different varieties sensors, including MCP9700 temperature sensor, DHT11 humidity sensor and pressure sensor. The lower power MCP9700 liner is a functional thermistor IC (microchip developed). A wide range of temperature measurements can be achieved between -40°C to 125°C when operated on a voltage value of 2.3volt to 5.5 volt. The DHT11 represents single wire sensor for communications that are typically used for both temperature humidity estimations temperature, with a lesser thermal range in temperature measurements than MCP9700. Lower data rate applications are enables by such single wire communication sensors.

**Supplementary Note 3 (Environmental IOT application Code sample):**

float UTCoffset = 0;

unsigned long lastMillis = 0;

unsigned long currentMillis = 0;

unsigned long secsSince1900 = 0;

bool daylightSavings = false;

bool hourTime = false;

int interval = 30000; //

String timeStr = "";

String webMessage = "";

String dateStr = "Temperature equals";

unsigned int localPort = 2390; // local port to listen for UDP packets

/* Don't hardwire the IP address or we won't get the benefits of the pool.

Lookup the IP address for the host name instead */

//IPAddress timeServer(129, 6, 15, 28); // time.nist.gov NTP server

IPAddress timeServerIP; // time.nist.gov NTP server address

const char* ntpServerName = "time.nist.gov";

const int NTP_PACKET_SIZE = 48; // NTP time stamp is in the first 48 bytes of the message

byte packetBuffer[ NTP_PACKET_SIZE]; //buffer to hold incoming and outgoing packets

// A UDP instance to let us send and receive packets over UDP

WiFiUDP udp;

// Initialize the OLED display using Wire library

SSD1306 display(0x3c, D3, D5);

dht11 DHT11;

/*-----( Declare Constants, Pin Numbers )-----*/

#define DHT11PIN D13

int humidity_val=0;

ESP8266WebServer server(80);

////////////////////////////////////////

//////// Sensors Value ///////////////

////////////////////////////////////

float temperature() // read temperature function

{

int ADVal, Tc_100, whole, fract;

ADVal = analogRead(0);

Tc_100 = 25 * ADVal - 2050;

// calibration

whole = Tc_100 / 100-20;

fract = Tc_100 % 100;

Serial.print("Temperature Value = ");

Serial.print(whole);

Serial.print(".");

if (fract < 10)

{

Serial.print("0");

}

Serial.println(fract);

delay(1);

return float(whole)+float(fract)/float(100);

}

void Humidity() // Single wire interface code to read humidity value

{

int chk = DHT11.read(DHT11PIN);

Serial.print("Read sensor: ");

switch (chk)

{

case 0: Serial.println("OK"); break;

case -1: Serial.println("Checksum error"); break;

case -2: Serial.println("Time out error"); break;

default: Serial.println("Unknown error"); break;

}

humidity_val= DHT11.humidity;

}

///////////////////////////////////////////////////////////////////////////

String getAJAXcode()

{

String webStr = "";

webStr += "<script src=\"http://ajax.googleapis.com/ajax/libs/jquery/1.10.2/jquery.min.js\"></script> \n";

webStr += "<script>\n";

webStr += " function loadTime() { \n";

webStr += " $(\"#timeDiv\").load(\"http://" + WiFi.localIP().toString()+ "/time\"); \n";

webStr += "} \n\n";

webStr += " setInterval(loadTime, 1000); \n"; // every x milli seconds

webStr += " loadTime(); \n";// on load

webStr += " </script> \n";

return webStr;

}

**Supporting Videos:**

Movie S1(a). Wing Slow Motion

Movie S1(b). Nature inspired H-TENG

Movie S2.PH level measurements
